# Framework for Armature-Based 3D Shape Reconstruction of Sensorized Soft Robots in eXtended Reality

**DOI:** 10.3389/frobt.2022.810328

**Published:** 2022-04-28

**Authors:** Elvis I. A. Borges, Jonas S. I. Rieder, Doris Aschenbrenner, Rob B. N. Scharff

**Affiliations:** ^1^ Department of Sustainable Design Engineering, Industrial Design Engineering, Delft University of Technology, Delft, Netherlands; ^2^ Department of Mechanical Engineering, Aalen University, Aalen, Germany; ^3^ Bioinspired Soft Robotics Laboratory, Istituto Italiano di Tecnologia, Genoa, Italy

**Keywords:** eXtended reality (XR), robotics, soft robot proprioception, 3D shape reconstruction, soft robot actuation, augmented reality, skeletal animation, teleoperation

## Abstract

Soft robots are typically intended to operate in highly unpredictable and unstructured environments. Although their soft bodies help them to passively conform to their environment, the execution of specific tasks within such environments often requires the help of an operator that supervises the interaction between the robot and its environment and adjusts the actuation inputs in order to successfully execute the task. However, direct observation of the soft robot is often impeded by the environment in which it operates. Therefore, the operator has to depend on a real-time simulation of the soft robot based on the signals from proprioceptive sensors. However, the complicated three-dimensional (3D) configurations of the soft robot can be difficult to interpret using traditional visualization techniques. In this work, we present an open-source framework for real-time 3D reconstruction of soft robots in eXtended Reality (Augmented and Virtual Reality), based on signals from their proprioceptive sensors. This framework has a Robot Operating System (ROS) backbone, allowing for easy integration with existing soft robot control algorithms for intuitive and real-time teleoperation. This approach is demonstrated in Augmented Reality using a Microsoft Hololens device and runs at up to 60 FPS. We explore the influence that system parameters such as mesh density and armature complexity have on the reconstruction's key performance metrics (i.e., speed, scalability). The open-source framework is expected to function as a platform for future research and developments on real-time remote control of soft robots operating in environments that impede direct observation of the robot.

## 1 Introduction

Soft robots are typically intended to operate in highly unpredictable and unstructured environments. Although their soft bodies help them to passively conform to their environment, the execution of specific tasks within such environments often requires the help of an operator that supervizes the interaction between the soft robot and its environment and adjusts the actuation inputs in order to successfully execute the task. However, direct observation of a soft robot is not possible when the robot is operating underground (Calderón et al., 2016), underwater (van den Berg et al., 2020), *in vivo* (Runciman et al., 2019), or in confined spaces (Takayama et al., 2015; Mazzolai et al., 2019). In such scenarios, the operator has to depend on a real-time simulation of the soft robot’s configuration based on the signals from proprioceptive sensors. Although several proprioceptive sensing and calibration methods for soft robots have been developed (Scharff et al., 2018; Van Meerbeek et al., 2018), the complicated three-dimensional (3D) configurations that are being predicted from this sensor data can be difficult to interpret using traditional visualization techniques. This ultimately leads to failures to complete tasks or inefficient task performance. A real-time 3D simulation of the soft robot on an immersive display [i.e., Extended Reality (XR)] would greatly support the operator in remotely controlling the robot. Besides providing an intuitive way of observing the soft robot configuration in 3D space, XR allows for the display of additional information (e.g., the locations and magnitudes of external forces acting on the robot extracted from additional embedded sensors) pertaining to the simulated soft actuator. Moreover, the XR environment could be linked to the soft robot control environment such that the real soft robot could be controlled using the virtual twin’s pose as a control input. However, it is currently unclear how the proprioceptive signals can be used to reconstruct the 3D shape of soft robots in extended reality in real-time. Therefore, this work addresses the following research question: How can the 3D shape of soft robots be reconstructed in real-time in XR?

**FIGURE 1:**
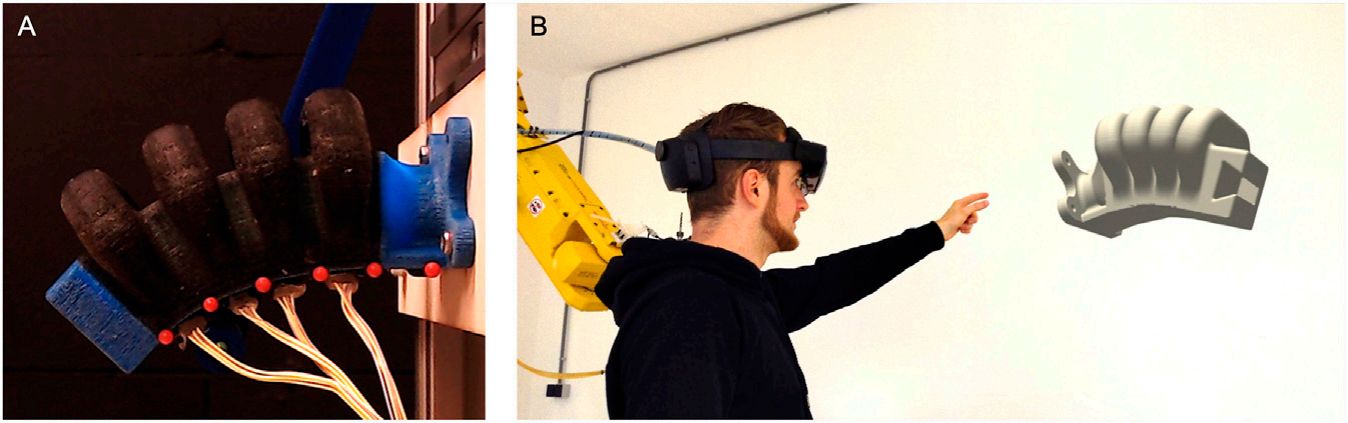
**(A)** Sensorized Soft Actuator with embedded color sensors for proprioception **(B)** A real-time digital twin of the deformed soft actuator in augmented reality based on the measurements from the proprioceptive sensors.

In this work, we present an open-source framework for real-time 3D shape reconstruction of soft robots in XR (Augmented Reality (AR) and Virtual Reality (VR)). The effectiveness of the proposed reconstruction framework is verified on a previously developed soft bending actuator with embedded proprioceptive sensors (Scharff et al., 2018 and Scharff et al., 2019). The signals from the embedded sensors are converted to parameters of a virtual armature which then controls a mesh representation of the soft actuator through the Unity skeletal animation framework. This functionality is enabled by a Robot Operating System (ROS) backbone that interacts with all components and allows for easy integration with existing soft robot control frameworks (McKenzie et al., 2017). The approach is demonstrated in Augmented Reality (AR) (Figure 1) using a Microsoft Hololens device and runs at up to 60 FPS. In addition, we explore the influence that system parameters such as mesh density and armature complexity have on the reconstruction speed and scalability. Side-by-side comparisons of the reference footage and the AR reconstruction show that our reconstruction accurately represents the deformation of the soft robot while it is interacting with its environment. The methods used by the framework and described in this paper can be divided into two clear phases: The pre real-time phase, which consists of model, scene, sensor and device adaptation and the real-time phase, which focuses on the methods that run to convert the sensor data into actionable bone and armature animation data for the AR visualization. The work is organized as follows: Section 2 covers the related work in extended reality for robotics, soft robot shape reconstruction, and skeletal animation. Section 3 describes the developed reconstruction framework in detail starting with the pre real-time subsection (section 3.3), followed by the real-time methods (section 3.4). The experimental setups used while testing this technique are briefly discussed in Section 4, while the framework’s performance is evaluated in section 5. Finally, section 6 will discuss conclusion and future work.

## 2 Related Work

In this chapter, we discuss the related work and review the different contributing fields combined in this publication. We start with eXtended Reality for Robotics before we address the Soft Robotics domain with respect to shape reconstruction. Finally we cover skeletal animation which is our approach for the 3D animation of the soft robot actuator.

### 2.1 Extended Reality for Robotics

Various studies (Lorenz et al., 2015; Ludwig et al., 2018) have shown that the productivity of future manufacturing facilities will be proportional to the ability of humans to communicate and interact with smart automated machinery and systems. With the growing complexity of robots and their workplaces (BCG Group, 2015), as well as their increasing cooperation with people, there is a need for advancements in the field of Human-Computer Interaction (HCI), more specifically in our case, in Human-Robot Collaboration (HRC) (Dianatfar et al., 2021). Extended reality (XR), with its sub-fields of augmented, mixed, and virtual reality (AR, MR, VR) offers a range of powerful tools and methods that can be utilized to simplify, augment and help in productive interaction between humans and robots. The implementation of XR in manufacturing has been proven feasible, such as AR-based repair instructions (Aschenbrenner et al., 2019) or displaying additional information within a factory (Peake et al., 2016). While significant research was conducted for each topic, HRC and XR, cross-technology research is yet less prominent with very few exceptions (Aschenbrenner et al., 2020). With the increasing popularity of using digital twins, XR can serve as a way to interface with a digital twin in the context of its intended physical environment. Applied for the HRC area, XR can enable a real-time link between robots and XR environments (e.g., Microsoft Hololens or an Oculus Quest). This increases the legibility of the robot and the situation awareness of the user. Multiple frameworks have been published (Hussein et al., 2018; Babaians et al., 2018; Aschenbrenner and Rieder, 2021) for such tasks. In this work, the MQTT-based DTStacks (Rieder, 2021) framework was chosen and customized for this application. This MQTT protocol choice is motivated in more detail in section 4, with the conclusion that it is the communication protocol most suited for real-time applications, and possesses several attributes beneficial for the particular functionality in this framework.

### 2.3 Soft Robot Shape Reconstruction

This subsection focuses on soft robot shape reconstruction based on proprioceptive signals. Proprioceptive sensing for soft robots is an active field of research that aims at developing sensors capable of capturing virtually infinite DOFs, large deformations, and large material strains without restraining the deformation of the soft robot. For an extensive overview of sensor technologies for soft robots, please refer to Wang et al. (2018).

After capturing the soft robot deformation, the shape of the robot needs to be reconstructed from this information. This is a challenging task that requires efficient parameterization of the soft robot shape. Commonly used shape parameterizations for calibrating soft robotic bending actuators are, for example, a single bending angle (Elgeneidy et al., 2018) or curvature (Zhao et al., 2016). Such models oversimplify the configuration space of the robot and are therefore incapable of accurately describing robot configurations that occur when the soft actuator is interacting with its environment. Alternatively, sensors have been calibrated to predict a number of predefined points on the robot (Thuruthel et al., 2019; Scharff et al., 2019). However, it is hard for an operator to visualize the complicated 3D shape of a soft robot based only on a set of 3D coordinate points. A representation of the soft robot by a set of 3D curves, such as a piece-wise constant curvature model (Jones and Walker, 2006; Della Santina et al., 2020), Cosserat rod model (Till et al., 2019), or Bézier surface (Scharff et al., 2021) helps the operator to interpret the shape of the robot, but cannot be used to visualize the appearance of the soft robot. The appearance of a soft robot can be reconstructed in real-time as a point cloud (Wang et al., 2020). However, this representation cannot be directly used for visualization in XR. This work focuses on soft robot shape representation through a triangle-based mesh to ensure compatibility with modern graphics engines. In the broader domain of shape reconstruction, certain computational techniques have been applied to reconstruct meshes from differing data sources, including 3D pointclouds (Yang et al., 2021, or 2D image inputs (Kolotouros et al., 2019; Nguyen et al., 2022)). Our method relies on skeletal animation to visualize the deformed soft robot shape from the sensor data in real-time.

### 2.4 Skeletal Animation

Skeletal animation was introduced in 1988 as a technique to deform arbitrary meshes to create animations by having a hierarchical “skeleton”—a set of kinematic joint nodes with associated rigid transforms (Magnenat-Thalmann et al., 1988). Skeletal animation remains the primary technique for real-time control of complex 3D meshes (alternative methods for mesh animation such as simulation based models (Terzopoulos et al., 1987; Sukumar and Malsch, 2006) while accurate, cannot provide real-time animation solutions, or cage-based methods (Nieto and Susín, 2013), which use a lattice-based structure to control vertices cannot handle complex deformations and present difficulties in hierarchical composition).

Both the translation between the skeleton bone transforms and the transforms that are applied to each of the mesh vertices to effect the mesh deformation are encoded in the skinning algorithm. There are several approaches in the literature for skinning metrics to deform meshes (Rumman and Fratarcangeli, 2016): whether through physics-based simplified mechanical simulations (Nealen et al., 2006; Sorkine and Alexa, 2007), or example-based methods (Mukai, 2018), such as motion matching, which uses interpolated example data from the real world or created by artists for the mesh deformations. Finally, we have geometric-based metrics, which use the skeleton transforms directly to compute the final vertex transforms. They are the standard skinning methods for interactive applications due to their relative simplicity and efficiency Rumman and Fratarcangeli, 2016).

This research project adopts a hybrid approach, where the use of these standard animation pipelines allows for real-time mesh deformations. Still, the animation is driven by an accurate estimation of the soft actuator’s configuration coming from its proprioceptive sensors. As a result of this, our approach effectively balances accuracy and efficiency.

## 3 Methods

This section starts with a broad overview of the entire pipeline, followed by subsections that go into further detail on the sensorized soft actuator, pre-processing steps and finally the real-time processing.

### 3.1 Overview

**FIGURE 2:**
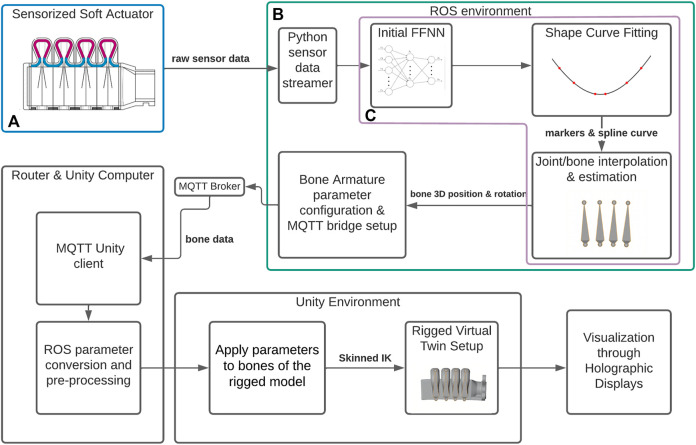
Schematic overview of the system framework that converts the measurements from a sensorized soft actuator **(A)** to a real-time 3D visualization through holographic displays. **(B)** denotes the ROS environment and the software nodes within, while **(C)** indicates the Real-time processing nodes of the framework.

Figure 2 shows a broad overview of the real-time framework. It starts in the top-left region (Figure 2A, highlighted in blue), where the actual physical actuator is deformed, through the air pressure differential in the bellows. The light sensors inside these bellows output the RGBC (red, green, blue, clear) data that is sent through USB to the Linux based ROS computer (in this case an Intel NUC). In the ROS environment (Figure 2B, in green) this raw sensor data is pre-processed and streamed through ROS to the next node, where it serves as the input of the first Feed-Forward Neural Network (FFNN), which converts this data into the marker coordinates, a process described in Scharff et al. (2019).

The next modules take these marker coordinates, and eventually convert them into the “bone” animation data used by the Unity system. Bones would link two joints in these “skeletons”. These are linked with the mesh vertices transforms, allowing for the mesh to be controlled indirectly through the set of joint transforms of the skeleton instead of the vertices individually. This process of creating and merging this skeleton is referred to as rigging, where each bone has a rigid 3D position rotation and scale, and influences a portion of the mesh it is linked to. There are three main steps in this process in our method: first, a spline curve is fitted on the markers, acting as a “main skeleton” of the current actuator deformation; followed by an interpolation step that locates the positions and orientations of the animation “bones” in the digital twin model of the actuator. Using higher-order derivative information from the interpolated spline function, the last step, packs these orientations into a single struct, such that the full actuator deformation is encoded in a way that can be streamed and decoded by the Unity script. While initially done explicitly, later on, these steps were encoded holistically through a second FFNN (Figure 2C, in purple). In summary, the pipeline is based on the following essential stages: Animated 3D model setup rig, Optical sensor data capture and processing, the animation reconstruction from the FFNN data to a set of bone configurations for the skeletal animation, and the live rendering of this data through the Unity interface.

This data struct is forwarded using Message Queing Telemetry Transport (MQTT), which connects the ROS sub-systems with the Unity environment on the HoloLens 2 *via* a (local) MQTT broker. Within Unity, the skinned, rigged 3D model’s bones are updated with every new received data message, allowing for a direct visualization of the model *via* the holographic displays. This was done nominally at up to 60 FPS. Concluding this overview, the framework has the following four requirements for reconstructing soft robots:

1) We possess a soft actuator with embedded (or external) real-time sensing feedback that can deliver data through serial ports to the ROS computer (section 3.2).
2) We possess a virtual twin of such an actuator, i.e., a 3D model that can be rigged to simulate the actuator’s DoFs (section 3.3).
3) We possess some method of embedding a mapping between this raw actuator data and the morphology of the actuator itself (in this work’s demonstration, we used the results of previous work (Scharff et al., 2018), where this is done through a marker based method, color sensors and a FFNN).
4) We possess a way of converting this morphology data into Mesh rigging/skinning data (subsection 3.4.1).

### 3.2 Sensorized Soft Actuator

**FIGURE 3:**
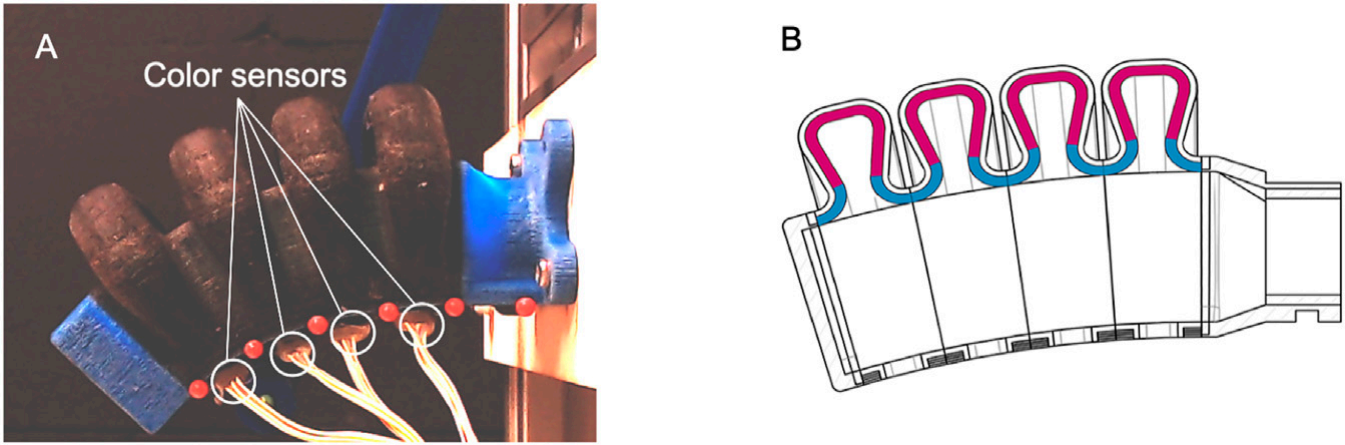
**(A)** Sensorized Soft actuator embedded with four color sensors and six red markers on the inextensible layer. **(B)** Illustration demonstrating the signal transducing color pattern inside the actuator’s air chamber.

The framework for real-time reconstruction of soft robots in XR is applicable to a wide range of sensorized soft robots. In this work, we demonstrate the framework on the sensorized soft pneumatic bending actuator shown in Figure 3A. The sensing principle is based on capturing 3D-printed color patterns inside the actuator’s air chamber (Figure 3B) with a small number of color sensors that are embedded on the inextensible layer of the actuator. Through a machine learning based calibration process, the change in color that is observed by the color sensors upon deformation was used to predict the relative 2D coordinates of six markers that were placed on the soft actuator. These primitive shape parameters form the basis for animating the rigged actuator 3D model. For more information on the sensing principle and calibration process, please refer to Scharff et al. (2018); Scharff et al. (2019). In this work, we make use of the open-access calibration and evaluation dataset (Scharff, 2021) that was generated by Scharff et al. (2019). The calibration dataset consists of the RGBC measurements of the four color sensors and the corresponding 2D marker coordinates for 1,000 different actuator configurations. The evaluation dataset consists of a video where the actuator interacts with a variety of objects in combination with the corresponding RGBC-measurements.

### 3.3 Pre-processing Steps

Before delving into the real-time framework, we must first begin with a brief discussion over the pre-processing steps that enabled such a pipeline.

**FIGURE 4:**
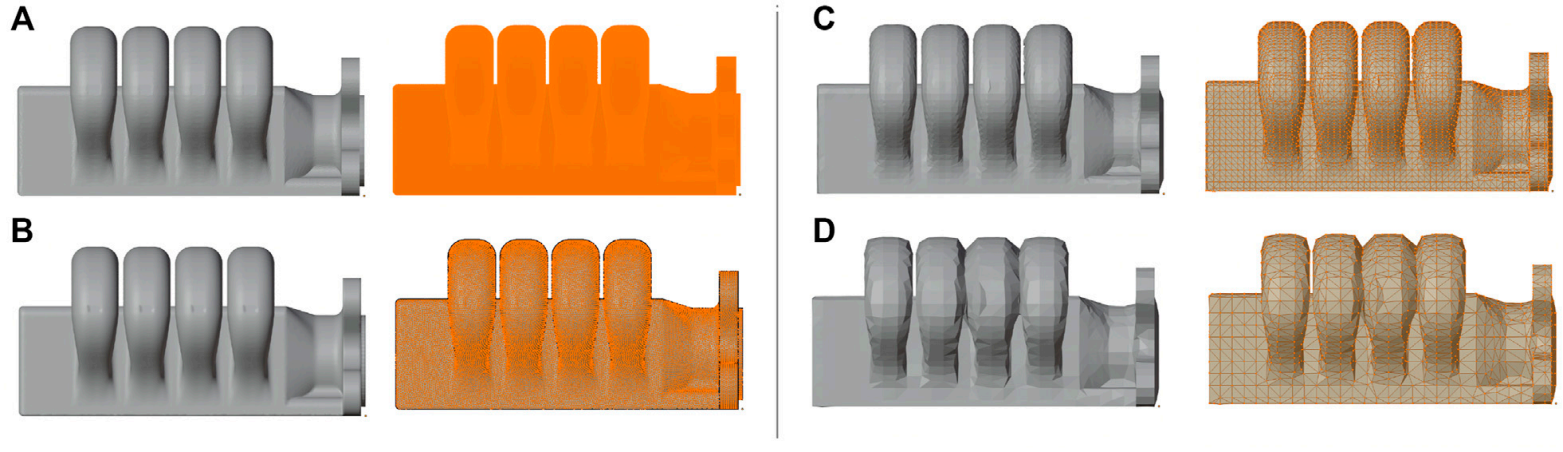
Different Sampling visualizations of original CAD model, with decreasing mesh density per row. The original cad model was exported from SOLIDWORKS, and in Blender, it was sampled at different densities [**(A)** 847 k verts, 1.7 M tris; **(B)** 82 k verts, 164 K tris; **(C)** 13 k verts, 26 k tris; and **(D)** 3.2 k verts, 6 k tris], after which the decimation/remeshing algorithms which simplified the mesh topology.

To have the previously mentioned digital twin of the actuator, we must start with the rigged 3D mesh. This was achieved through the blender toolset (Blender Community, 2018). Before the animation steps, the digital twin’s model needs to be simplified, as the CAD fabrication one possesses too much detail. As mentioned previously, the fabrication model is resampled in blender. This resampling provides a mesh which is clustered at the surface, at different densities. To optimize the mesh density, consecutive rounds of decimation and re-triangulation algorithms are applied, until we end at a topology that maximizes detail fidelity with minimal vertex count (as can be see in Figure 4, where (Figures 4B,C) present the configurations that best encoded these desired characteristics).

We use the animation standard skinning method, Linear Blend Skinning, which is a direct geometric-based method (Lewis et al., 2000). To animate and deform the skeleton bone set from one “pose” to another, where each mesh vertex position will depend on the following basic equation:

$$v_{i}\left(t\right)=\overset{n-1}{\underset{j=0}{\sum }}w_{ij}B_{j}\left(t\right)v_{{\mathrm{ref}}_{i}}$$ (1)

**FIGURE 5:**
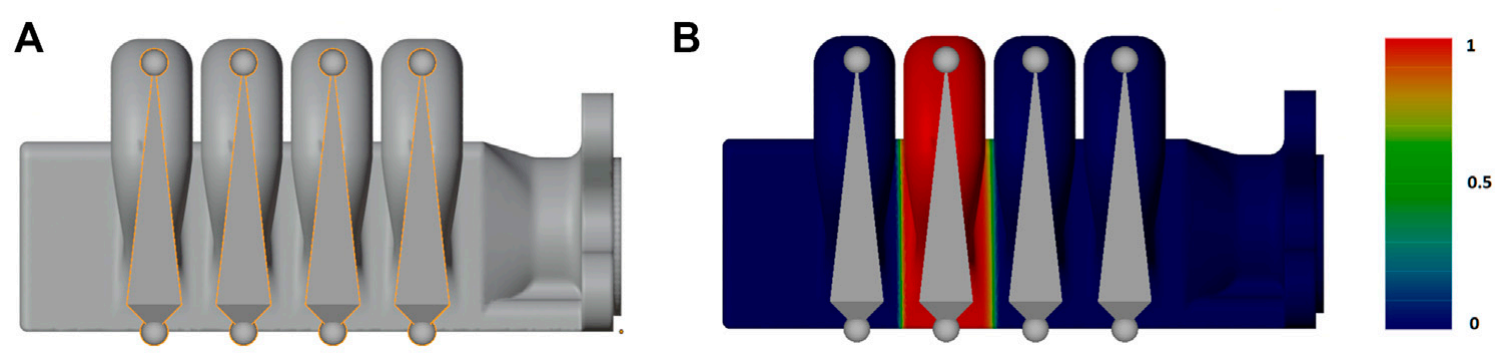
**(A)** Rigged simplified 3D actuator model, along with **(B)**, showing the weight painting gradient for the third bone [from red (maximum vertex influence) to dark blue—zero influence] for one bellow and its corresponding bone. This gradient is a visual representation of the vertex weights set for this mesh for the third bone (Scharff et al. (2018))

Note that a skeleton’s pose is always defined in reference to the skeleton at its initial pose, in our case, the actuator when all the bellows are non-actuated. A given pose will move all the mesh’s vertexes from this initial pose position $\left({v_{ref}}_{i}\right)$ to *v*_*i*_. In this mathematical model, given a tree of joints that compose the skeleton, and the bones that link two joints and manipulate them, each vertex ***v***_***i***_’s position in relation to a specific time (**t**) will depend on a linear combination of several deformation primitives. Namely, $B_{j}\left(t\right)\in R^{3\times 4}$ , the relative transform of bone *j* encodes the transform of the associated joint *j*, relative to the reference pose. *w*_*ij*_ is the set of skinning weights. This set exists because a single vertex can be influenced by several bones. The sum of this set per vertex over all of its associated bones is unitary $\left(\sum_{j=0}^{n-1}w_{ij}=1\right)$ . The specific partition of this set is done by the skinning algorithm. In our case, a particular bone’s influence on a vertex is encoded in the weight painting step (Figure 5B).

Rigging the 3D model was a manual process, the actuator is composed of a soft material section, with two hard 3D-printed parts at the end. As this “finger” actuator’s main DOF (Degree of Freedom) is the contraction/extension of the bellows through the air pressure, and the bellows’ expansion is simultaneous, the rigging skeleton is simple, a bone can control the position of each bellow segment, with both ends of the actuator being connected to the soft actuator section through fixed constraints. Each bone has a fixed reference between each-other, as they are perfectly centered in each bellow segment. The bone’s final position is parameterized with the marker’s coordinate position information, which we obtain from the animation processing pipeline from the FFNN.

With the actuator design, the skinning approach was simple, using a gradient ramp applied to the bones, such that each bone influences its corresponding bellow, with the exception of the base bone, which is attached to the actuator’s base.

### 3.4 Real-Time Processing

#### 3.4.1 Animation Processing From Optical Sensor Data

The starting point for representing the correct bone coordinates and rotations (and thus the global deformation of the actuator) are the coordinates of the six markers in the in-extensible layer of the actuator. Each bellow segment of the actuator possesses a color sensor embedded in the actuator’s in-extensible layer. The first FFNN takes the RGBC color sensor data as inputs and outputs the six marker coordinates (X, Y, in millimeters). It is composed of two-layers, with a hidden layer (with a sigmoid transfer function) with ten neurons, with the output layer using a linear transfer function. The 16 raw sensor inputs then become 12 coordinate outputs.

The final animation skeleton pose requires the 3D position (relative to the actuator origin i.e, the base marker or the attachment part) and rotation of each bone, thus each skeletal pose requires 30 inputs. This pose struct, is sparse due to the inextensible material constricting the soft bellows (e.g., the base spine layer the markers are deposited into). The bellows’ 3D position can be retrieved with its delimiting marker’s (X,Y) coordinate relative to the origin, and the bellow’s angular orientation relative to its “rest” pose (Figure 3A), where the deviation is 0°, as the markers ground truth rest coordinates are known a priori. As each bone is perfectly centered between its delimiting “base” markers, its orientation and (X,Y) origin coordinates can be obtained by defining a differentiable interpolated piece-wise cubic spline curve from the marker coordinates. This piece-wise polynomial connects the marker positions with third degree splines. The smooth behavior of this approximated spline means that N-dimensional derivative information for this curve is available, for any point sampled from it. Each bone’s origin position is sampled from the parameterized curve location (in relation to the actual distances in the real actuator), and the bone’s normal orientation is obtained through the rotated tangent technique. With this technique, given the bones origin position along the curve, the normal vector $\left(\hat{n}\right)$ to this point can be estimated by the rotated normalized tangent vector at that point in the curve itself.

This tangent vector is calculated by first obtaining the first derivative at the bone’s location along the parametric form of this curve $\frac{d\vec{f}\left(bone_{i}\right)}{dt}$ (the t parameter encodes the position along the curve this tangent is calculated at). As the normal vector is orthogonal to this tangent one, it can be obtained by a simple positive 90° 2D rotation. Once this normal vector is normalized, the bone’s angular orientation can be retrieved by calculating its dot product with the reference coordinate vector of a bone at rest angular position $\left(\hat{j}\right)$ : $\hat{n}\cdot \hat{j}=|\hat{n}\|\hat{j}|\cos\left(\theta \right)\equiv \theta =\arccos\left(\hat{n}\cdot \hat{j}\right)$ , given that the vectors are basis vectors (|*v*| = 1).

Alternatively, these transformations can be encoded end-to-end. After validating the previous approach for the actuator visualization, a new FFNN was devised, with the same structure as the initial one, except for the output layer, which has 14 nodes (the fixed pose parameters, such as the origin are excluded). While the full skeletal pose takes 30 parameters, the DOF constraints inherent in the prototype design allow these parameters to be fixed. This new network was trained using the Bayesian Regularization algorithm, using a standard division of end-to-end samples (70% for training—15% for validation set, 15% for testing).

#### 3.4.2 Rendering in Augmented Reality

For the final step in this framework, this skeleton animation data is sent over a local network to a (local) MQTT broker and forwarded to the Hololens 2 running the real-time unity simulation environment. This consists of a scene with the rigged 3D model in which a C# script updates each bone’s parameters, given a new message from the broker, to compose a final skeletal pose of the actuator. To prevent any visual artifacts, and smooth out temporal glitches, the final poses between subsequent frames are linearly interpolated (as a free parameter of the visualization). The Hololens 2 then renders the latest bone positions and rotations (3D movement and rotation) to visualize the model in real-time.

## 4 Experimental Setup

As alluded to previously, several tools are used in this real-time visualization technique. These can be divided in roughly two stages: First, interfacing with the actuator, capturing the data and converting it into the animation pose, and second, animating the rigged actuator virtual twin in an AR scene setup. We used an Intel NUC computer, which used the Robot Operating System (ROS) to collect data from the sensor (through serial port) in a node, and transfer it to the animation ROS node, which implemented the FFNN and created the animation pose data. For benchmarking the alternative methods under similar constraints repeatedly, raw actuator data was collected previously under set constraints into a database, which was then fed directly to the rest of the framework. MQTT-Bridge GROOVE-X (2020), was set-up in the system, which, with the help of a router, fed the animation poses through the local network, *via* a (mosquitto) MQTT Broker, to the HoloLens 2. The HoloLens 2 received the MQTT messages *via* a customized version of the MQTT framework “DTStacks” (Rieder, 2021). This MQTT protocol originated in the IoT (Internet of Things) field. It was chosen due to the fact that it is an industry standard protocol, with broad device/platform support (from web hosts to embedded devices)—allowing for maximum flexibility in connecting the portions of our framework and for future work with minimum user effort, i.e., there are easily available plugins handling communication in all of different modules of the framework, requiring no extra work for the connection overhead. Its implementation is simple, and it is a data agnostic, lightweight and bandwidth efficient way of transferring data wirelessly. MQTT is also optimized for low latency, thus being more suited for real-time applications, furthermore it is open source, light-weight to be used in a Game engine for a mobile device and can use global addresses. The actuator model was staged in a fixed location and manipulated based on the latest received data struct, with each received message containing all relevant information for all joints.

For preparing the networks and trigger the tests, the dataset was obtained through a setup in which the live actuator would be actuated with a randomized amount of pressure, and the position of an obstacle. At this point, both the sensor’s state and the ground-truth deformation were captured with a camera, with the ground truth being obtained from extracting the marker’s position from the calibrated images. More details on the conditions of this dataset construction can be found in Scharff et al. (2019).

## 5 Results

The open-source framework for real-time 3D shape reconstruction of soft robots in XR can be accessed on the 4TU Research Data Repository (Borges and Rieder, 2021). The framework includes ROS code for interfacing with the actuator, together with Unity scripts that allow for live animation data to be visualized in AR. The code is accompanied by an extensive documentation that allows others to easily set up the framework as well. To allow others to compare results on future implementations of the proposed framework, the image/video-data and the corresponding sensor values that were used for calibrating the soft actuator and evaluating the framework have been made available online as well (Scharff, 2021). The framework can achieve through puts of 66 FPS. The Hololens 2 AR device requires application developers to maintain 60 FPS, giving a 16 ms latency budget for the framework to send a new pose to Unity. The demo presents a low case of complexity scene, as there are no textures applied to the soft robot’s virtual twin, with around 100 k vertices. Thus, on the application side, the only performance bottleneck that remains are the post-processing filters (such as shadows/lighting). This provides an uniform stage for testing the performance of the framework itself.

### 5.1 Evaluation and Comparisons

Starting with Linear Blend Skinning (LBS), the amount of required operations is connected to two main factors: mesh density, and bone complexity.

**FIGURE 6:**
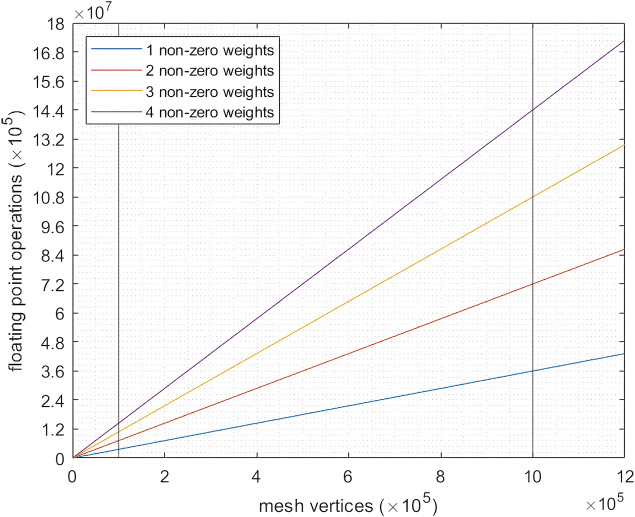
Floating point operations per pose needed for a skinned mesh using the default blender animation pipeline, in relation to armature complexity (number of related bone-pairs to a single vertex), with highlighted comparisons between a mesh requiring 100 k vertices vs. one with 1 M vertices, with the assumed industry standard of a maximum of four associated bone-pairs per vertex.

There are two main bottlenecks to the animation performance in this setup 1): the number of bones of a given skeleton, as a vertex may use the transformation matrix data of several bones of the skeleton (through the weight parameter set), and the number of mesh vertexes that must be transformed (this is due to the rendering process being dependent on the mesh vertices). As a linear operation (Figure 6), retaining a simple scene setup is direct. By reducing mesh density with decimation algorithms and restraining related bone pairs to specific vertices (ideally, a single one), the number of parameter updates for a new pose of the mesh can be restrained (this is not always possible, as complex deformations cannot be faithfully replicated with a single bone influence per vertex). Due to the 3D-printed process involved in manufacturing the actuator, a CAD model was already available. Visualization does not require the vertex density necessary for fabrication (linearly correlated to animation performance) and, as in the skinning process only the external surface mesh is necessary, this raw converted mesh from the CAD model was simplified using mesh decimation techniques (Gotsman et al., 2002), namely vertex clustering around the external 3D surface, and decimation of the original mesh, from ∼1 M vertexes to approximately 100 k. As can be seen in Figure 4B, the soft-robot behavior could not be replicated with the lowest fidelity mesh, as the overlap between the bellows renders the bone-vertex armature unfeasible, as the bellow transitions required a vertex to be influenced by the adjacent bones as well, though these vertexes remains a minority of the full mesh. While further optimizations could be applied, these transformations already allowed for the reduction of necessary operations by a factor of 12, with these parameters being adjustable to a particular scene/actuator setup, whether towards more fidelity or more performance.

For the animation pipeline, the largest latency delay is in the animation processing phase. The total framework takes roughly (5.18 ± 1.42) ms to send new pose information to Unity. The neural network forward pass requires (0.68 ± 0.09) ms, with the spline and bone reconstruction section taking up (3.30 ± 1.05) ms. By replacing these two steps into an holistic FFNN this whole stage takes (2.69 ± 0.10) ms; however, such a structure has drawbacks in terms of robustness. Structuring the output data and sending it through the MQTT connection takes (1.19 ± 0.31) ms, with the animation script in unity’s side taking up $9.62\pm \left.0.38\right)\;ms$ , allowing for a best scenario system framerate of around 65.88 FPS^1^.

Unity’s Hololens performance guidelines provide certain boundaries in scene complexity, for keeping a table framerate. LBS is a linear algorithm, meaning that a given skeleton’s animation performance is linearly correlated between its number of vertices, with multiplier influence factor relying on armature (and thus, bone) complexity. While the influence matrix is sparse (due to the industry standard of maximum four bones connected to a single vertex), the exact factors will depend on the percentage of vertices in the skinned mesh that are influenced by one or more bones. In our case, the simple actuator possesses few multi-influence areas (essentially bellow wall gaps). Rendering several of these actuators in a scene is not limited to the animation pipeline section of the framework—ROS modular nature simply means an animation node can be instanced for each actuator, effectively parallelizing these, though this might add I/O overhead, depending on the available I/O ports. In the hololens environment, this is not easily adapted however, the linear complexity of the animation in regards to vertex count, means a scene with ten animated digital twins would have roughly a 10× performance decrease. However, this could be remedied by using lower fidelity meshes, (i.e., through decimation algorithms) though this has clear limits (e.g., the used actuator remains unaffected up to a ∼2.5× vertex decimation, after which serious mesh resolution deformations come to the fore).

**FIGURE 7:**
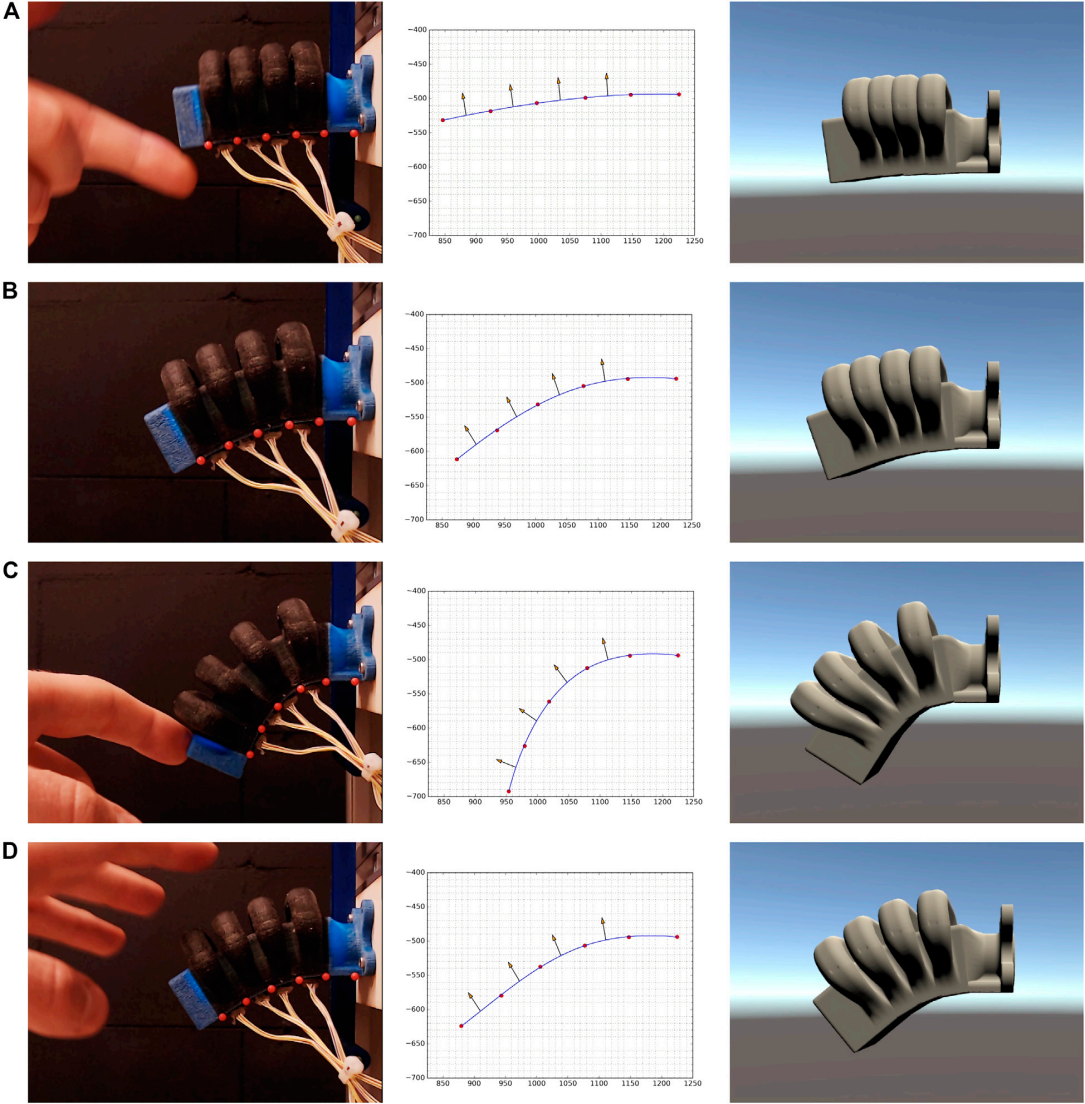
Demonstration of the proposed framework for a soft bending actuator. The first column shows the soft actuator while different actuation pressures and external forces are being applied. The second column shows the marker coordinates that are predicted from the sensor measurements, and the spline and bone vectors that are computed from these coordinates. The virtual twin of the soft actuator in the Unity environment is shown in the third column.

Finally, we may compare the final visualization with the expected marker positions and ground truth images (Figure 7). Beyond the base FFNN model’s accuracy, which is evaluated in more detail in previous work (Scharff et al., 2018), we can see that as the deformations get more extreme, the reconstruction becomes less faithful. This is due to several reasons, among them, the fact that the completely “inextensible” layer is a simplification, the gap that exists between the skinning “model” of the actuator and its actual deformation properties, and the innate error of the FFNN (0.0094 MSE between the expected results vs. the NN outputs). The main advantages of these networks lies in their fixed performance and good approximation behavior. In our case a simple FFNN with only 338 k parameters could replicate the bone estimation phase from the raw sensor data, at a loss of robustness, but do it predictably.

A video of the simulation synced with the footage of the real soft actuator is made available with the article.

## 6 Conclusion

The main research contribution of this work lies in devising a scalable, performant method for reconstructing a soft robot’s shape in real-time in a 3D environment. The proposed method takes advantage of standard real-time techniques used in animation and computer graphics, allowing for modern graphics tools.

The open-source framework is expected to function as a platform for future research and developments on real-time remote control of soft robots in environments that impede direct observation of the robot. Moreover, we believe the framework could be a valuable tool for other researchers to study a soft robot in their intended environment in order to identify possible areas for improvement.

In this work, the framework is demonstrated on a soft actuator that only bends in-plane. However, the approach can be easily extended to three-dimensionally deforming soft robots by fitting a 3D spline to several points on the robot. However, the approach does require that the shape of the soft robot can be approximated by an armature. This is the case for most commonly used soft robot designs, such as soft continuum arms, soft bending actuators, artificial muscles, and soft robots designed for peristaltic locomotion. The approach is applicable to any type of proprioceptive sensors. However, the sensors should be calibrated to ground truth data that can be easily converted to an armature (e.g., using strategically placed markers such as in this work).

For minimum user overhead, we believe marker/geometric based methods give a good balance of these considerations, as a fixed marker based construction is demonstrated to give good results for FFNN model-based actuator morphology predictions (Scharff et al., 2018). This marker/geometric-based method can be more simply parametrized to the Bone/Armature standard, as demonstrated in this work, with the performance and high throughput needed for real-time (as evaluated in section 5.1). Using such an approach for other soft robots consists mainly in retraining the FFNN model and adjusting the bone estimation method according to the new digital twin, and setting up the XR scene according to the desired scenario.

Future work will focus on displaying additional information about the environment or state of the robot in the simulation (e.g., location of the contact or the stress distribution). Moreover, future work could focus on the integration of not only simulation but also soft robot control through ROS (an example of soft robot control through ROS is demonstrated in McKenzie et al. (2017)). This integration could enable interaction between the soft robot and its virtual twin. For example, whereas the soft robot currently drives the reconstruction, it could also be controlled using the virtual twin’s pose as a control input.

Finally, the framework could be further optimized by automating the skinning and rigging steps, reducing visual glitches occurring from the mesh decimation phases, and simplifying the framework overhead to allow for more complex and realistic visualizations (complex lighting, shadows, multi-material soft robots).

## Data Availability

The datasets generated and analyzed for this study can be found in the 4TU Research Data Repository. The framework for 3D shape reconstruction of soft robots in XR (Borges and Rieder, 2021) can be accessed through the following link: https://data.4tu.nl/articles/software/Soft_Gripper_AR_Framework/16943254. The image/video-data and the corresponding sensor values that were used for calibrating the soft actuator and evaluating the framework Scharff (2021) can be accessed through the following link: https://data.4tu.nl/articles/dataset/Sensorized_Soft_Actuator_Datasets/16943239.

## Footnotes

1 Unity contains a certain latency delay. The bone data is only updated with the following frame. Meaning we have a rendering delay of up to 16 ms, however this “shift” is not noticeable for a user.

## Supplementary Material

Supplementary Video S1 Side-by-side comparison of the sensorized soft actuator and its virtual twin in augmented reality.
